# Effects of Integrative Autism Therapy on Multiple Physical, Sensory, Cognitive, and Social Integration Domains in Children and Adolescents with Autism Spectrum Disorder: A 4-Week Follow-Up Study

**DOI:** 10.3390/children9121971

**Published:** 2022-12-15

**Authors:** Yunhwan Kim, Mikyoung Kim, Chanhee Park, Joshua (Sung) H. You

**Affiliations:** 1 Sports Movement Artificial Robotics Technology (SMART) Institute, Department of Physical Therapy, Yonsei University, Wonju 26943, Republic of Korea; 2 Department of Physical Therapy, Yonsei University, Wonju 26943, Republic of Korea

**Keywords:** autism rehabilitation, children and adolescents with autism spectrum disorder, ICF model, integrative autism therapy, multiple domains

## Abstract

This study aimed to compare the effects of conventional autism therapy (CAT) and integrative autism therapy (IAT) in children and adolescents with autism spectrum disorder (ASD). A convenience sample of 24 children with ASD was recruited and underwent either CAT or IAT for 60 min/day, twice a week, for 20 sessions over 10 weeks. Outcome measures included the following: (1) physical domain (pediatric balance scale, PBS), (2) sensory domain (short sensory profile), (3) cognitive domains (functional independence measure, FIM; and childhood autism rating scale), and (4) social integration domain (Canadian occupational performance measure, COPM; short falls efficacy scale; and pediatrics quality of life questionnaire). Two-way repeated analysis of variance (ANOVA) was used to determine the intervention-related changes in the four domains across the pre-test, post-test, and follow-up test at *p* < 0.05. ANOVA showed significant interaction effects on the PBS, FIM, and COPM (*p* < 0.05) variables. Moreover, time main effects (*p* < 0.05) were observed in all four domain variables, but no group main effect was noted. This study provides promising evidence that IAT is more effective than CAT for managing children and adolescents with ASD.

## 1. Introduction

Autism spectrum disorder (ASD) presents with multiple domains of functional and structural impairments, physical activity limitations, and associated social participation and integration restrictions, affecting 1 in 44 children in the United States [1]. Four major affected domains have been identified: (1) physical (mobility, strength, and balance) [2,3]; (2) sensory (tactile, visual, and auditory sensitivity) [4,5]; (3) cognitive (self-care, locomotion, communication, and social cognition) [6]; and (4) social integration (performance, satisfaction, fear of falling, and quality of life) [7,8]. Unfortunately, such dysfunction is also correlated with an increased risk of other psychiatric and neurodevelopmental disorders [9], an adjustment in physical health [10], lower life satisfaction [11], and early death [12]. Presumably, such impairment domains are associated with abnormalities at the genetic, cognitive, and neural levels as well as with environmental factors [13,14,15].

Considering the various chronic neurodevelopmental problems associated with ASD and the limitations of currently available interventions, an unambiguous public need exists for effective and sustainable interventions. The birth of a child with ASD requires the family to revise their roles and reconsider their tasks and activities [16]. Throughout the life of the child, families must cope with new situations that bring new challenges and fresh responsibilities. In one study, the majority of the parents surveyed stated that the information necessary to understand their child’s ASD was seldom supplied directly; rather, it had to be expressly asked for [17]. Moreover, the information was often incomplete, not up to date, and lacking in terms of information about the consequences and likelihood of intervention in the short, medium, and long terms [18]. Focusing only on the child or adolescent with ASD is not enough; promoting family cohesion and positive relationships between parents as well as between parents and their children is also necessary [19]. Assessment activities should not privilege impairment, activity limitation, and participation restriction; they should also carefully consider parental resources and knowledge, as well as the abilities of family members to care for a child with ASD and cope with their experiences. Problems such as family isolation, reduced social networks, family cohesion, and failure to use social skills that are useful for encouraging positive and responsive relationships should alarm service providers and alert them to the need to intervene [20].

Conventional ASD therapy (CAT) includes physical and occupational therapies and behavior modifications (e.g., applied behavior analysis [ABA]); however, CAT produces variable results. Physical therapy focuses on physical domains to facilitate the acquisition of deficient motor abilities in static and dynamic conditions and to reduce the physical activity limitations presenting with ASD [2,21]. Physical therapy for ASD includes physical activity [22], hippotherapy [23], motor skill learning [24], and aquatic therapy [25] and has been reported to be effective in improving balance, postural control, and muscle coordination. Occupational therapy for ASD entails sensory integration therapy (SIT), sensory processing [26], and play therapy [27], which are beneficial for mild to moderate improvements in sensory and cognitive domains by stimulating tactile, vestibular, and proprioceptive systems to improve muscle tone, automated reactions, and emotional stability [28]. ABA is also widely utilized for psychological and emotional behavior modifications in children and adolescents with ASD [29]. ABA focuses on social and environmental modifications to make changes in ASD behavior, and studies [30] have demonstrated improvements in communication [31] and social skills [32]. However, CAT has primarily emphasized individual domains or components of multiple domains (i.e., physical, sensory, cognitive, or social integration domains) and International Classification of Functioning, Disability, and Health (ICF) levels (i.e., impairments, activity limitations, and participation restrictions) rather than addressing the integrative elements of ASD’s multiple domains and levels and their clinical ramifications [33]. As such, CAT tends to have a limited therapeutic effect on social integration and participation, which are most important for independent daily activities and associated quality of life.

To mitigate the shortcomings of the CAT framework, we have developed an integrative autism therapy (IAT), which is based on a conceptual scheme addressing the integrative therapeutic elements of multiple domains (i.e., physical, sensory, cognitive, and social integration domains), outcome measurement levels (i.e., impairments, activity limitations, and participation restrictions), and each family’s or client’s individualized goals. Specifically, the IAT utilizes the Canadian Occupational Performance Measure (COPM) to identify the individualized and specific demands of children and adolescents with ASD and their parents. Furthermore, interventional approaches have been personalized for the needs of children and adolescents with ASD and for their developmental goals to cover the multiple domains (i.e., physical, cognitive, sensory, and social integration domains). Therefore, this study aimed to compare the effects of CAT and IAT on ASD’s physical, sensory, cognitive, and social integration domains. Our study assessed functional balance using the pediatric balance scale (PBS) and sensory processing patterns using the short sensory profile (sSP) for the physical and sensory domains, respectively, in children and adolescents with ASD. In addition, we assessed activities of daily living using the functional independence measure (FIM) and autistic level using the childhood autism rating scale (CARS) for the cognitive domain. For the social integration domain, satisfaction and performance were assessed using the COPM, fear of falling using the Short Falls Efficacy Scale (sFES), and quality of life using the Pediatric Quality of Life Inventory (PedsQL™). We hypothesized that IAT would provide more beneficial effects on the physical, cognitive, sensory, and social integration domains than would CAT in children and adolescents with ASD.

## 2. Materials and Methods

### 2.1. Participants

A total of 24 children with ASD (girls = 6, mean age = 8.53 years) were recruited from the local community rehabilitation center and welfare center. The study was approved by the Research Ethics Committee of Yonsei University Mirae Campus (1041849-202009-BM-140-04), Republic of Korea, and was registered with the International Clinical Trials Registry Platform (KCT0006417). The parents of the participants signed a consent form before participation. The inclusion criteria were as follows: ASD diagnosis, age < 18 years, >30 points on the CARS for ASD, and the ability to follow instructions. The exclusion criteria were as follows: severe cognitive or visual impairments, cardiopulmonary system impairments, epilepsy or taking anti-epileptic drugs, and a history of surgery or trauma within the last year.

### 2.2. Research Design

**Figure 1:**
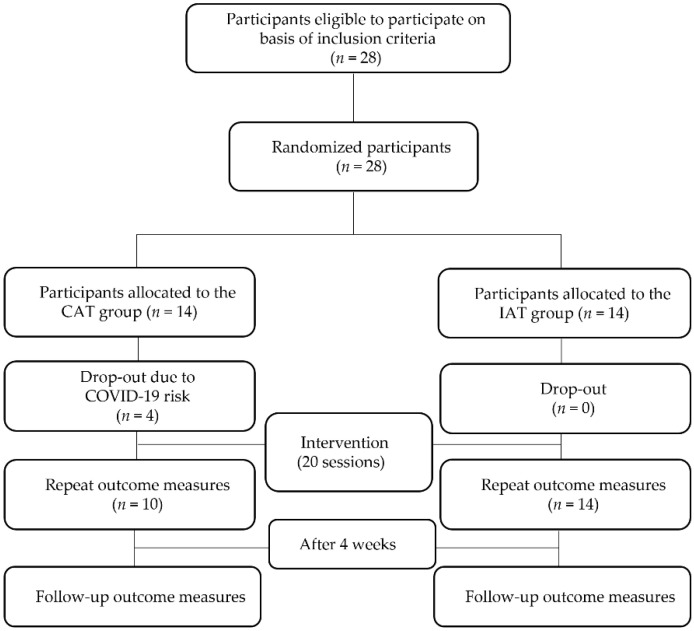
Flow chart.

A randomized, single-blind, experimental design was used in the present study. Twenty-eight participants were randomly assigned to either the CAT or IAT group via a random number generator in Excel software (Microsoft Corp., Remond, WA, USA). A researcher generated the random allocation sequence, another researcher assigned participants to interventions, and blinded researchers assessed outcome measures. To remove experimental biases associated with the participants’ expectations, experimental information that could affect the participants was masked until the experiment was completed. At the beginning of the study, there were 14 participants in each group; however, 4 participants dropped out of the control group. The underlying reason for this was an end to the intervention under the policy of the control group’s welfare center or a request by the guardian because of the threat of coronavirus disease 2019 (COVID-19). Standardized clinical testing procedures included the physical domain (PBS), sensory domain (sSP), cognitive domain (FIM and CARS), and social integration domain (COPM, sFES, and PedsQL). These measurements were consistently implemented during the pre-tests, post-tests, and follow-up tests. A flowchart of the study is shown in Figure 1.

### 2.3. Clinical Outcome Measurements

#### 2.3.1. Pediatric Balance Scale

The PBS assesses the motor function-oriented balancing ability of children and adolescents with impairments in the ICF model for the body structure and function domains. The test included 14 items ranging from the seated position to standing on one foot. The test scores ranged from 0 (“low function”) to 4 (“highest function”), with a maximum score of 56 points. The intra- and inter-tester reliability of PBS is well-established with intra-class correlation coefficient (ICC) = 0.97 and 0.98, respectively [34]. Specifically, the PBS 14 items include: “1. Sitting to standing; 2. Standing to Sitting; 3. Transfers; 4. Standing unsupported; 5. Sitting unsupported; 6. Standing with eyes closed; 7. Standing with feet together; 8. Standing with one foot in front; 9. Standing on one foot; 10. Turning 360 degrees; 11. Turning to look behind; 12. Retrieving object from floor; 13. Placing alternate foot on stool; 14. Reaching forward with outstretched arm” [34].

#### 2.3.2. Short Sensory Profile

The sSP assesses sensory processing dysfunction in children and adolescents with impairments in the ICF model for the body structure and function domains. The sSP consists of 7 subsets of sensory processing function tests (taste, tactile, smell, movement, visual, and auditory sensitivity and under-responsiveness/sensation seeking), totaling 38 items. The form responses ranged from 1 (“always”) to 5 (“never”) and were associated with child behaviors. The maximum sSP score was 190. The reliability and validity of the sSP have been previously identified, with ICC = 0.88 and r = 0.87, respectively [35]. Specifically, the sSP 38 items include: “1. Express distress during grooming (for example, fights or cries during haircutting, face washing, fingernail cutting); 2. Prefers long-sleeved clothing when it is warm or short sleeves when it is cold; 3. Avoids going barefoot, especially in sand or grass; 4. Reacts emotionally or aggressively to touch; 5. Withdraws from splashing water; 6. Has difficulty standing in line or close to other people; 7. Rubs or scratches out a spot that has been touched; 8. Avoids certain tastes or food smells that are typically part of children’s diet; 9. Will only eat certain tastes; 10. Limits self to particular food temperatures/textures; 11. Picky eater, especially regarding food textures; 12. Becomes anxious or distressed when feet leave the ground; 13. Fears falling or heights; 14. Dislikes activities where head is upside down; 15. Enjoys strange noises/seeks to make noise for noise’s sake; 16. Seeks all kinds of movement and this interferes with daily routines; 17. Becomes overly excitable during movement activity; 18. Touches people and objects; 19. Doesn’t seem to notice when face or hands are messy; 20. Jumps from one activity to another so that it interferes with play; 21. Leaves clothing twisted on body; 22. Is distracted and has trouble functioning if there is a lot of noise around; 23. Appears to not hear what you say; 24. Can’t work with background noise; 25. Has trouble completing tasks when the radio is on; 26. Doesn’t respond when name is called but you know the child’s hearing is OK; 27. Has difficulty paying attention; 28. Seems to have weak muscles; 29. Tires easily, especially when standing or holding particular body position; 30. Has a weak grasp; 31. Can’t lift heavy objects; 32. Props to support self; 33. Poor endurance/tires easily; 34. Responds negatively to unexpected or loud noises; 35. Holds hands over ears to protect ears from sound; 36. Is bothered by bright lights after others have adapted to the light; 37. Watches everyone when they move around the room; 38. Covers eyes or squints to protect eyes from light” [35].

#### 2.3.3. Functional Independence Measure

The FIM assesses the quality of daily living activities of children and adolescents with limitations in the ICF model for the activity domain. The FIM comprises 18 items, with subscales for self-care, social cognition, transfers, locomotion, communication, and sphincter control. Each item is responded to on a 7-point scale ranging from 1 (“complete dependence on the helper”) to 7 (“complete independence with no helper”). The reliability and validity of the FIM were previously reported, with ICC = 0.99 and Kappa coefficient = 0.92, respectively [36,37]. Specifically, the FIM 18 items include: “1. Eating; 2. Grooming; 3. Bathing; 4. Dressing—upper body; 5. Dressing—lower body; 6. Toileting; 7. Bladder management; 8. Bowel management; 9. Bed, chair, wheelchair; 10. Toilet; 11. Tub, shower; 12. Walk/wheelchair; 13. Stairs; 14. Comprehension; 15. Expression; 16. Social interaction; 17. Problem solving; 18. Memory” [36].

#### 2.3.4. Childhood Autism Rating Scale

CARS is a subjective, diagnostic assessment measurement in the ICF model for the activity domain that examines individuals on a scale ranging from non-autistic to severely autistic. CARS consists of 15 items and provides a comparison of affected children’s motor skills and behaviors with those of healthy children. Each item ranges from 1 (“normal behavior”) to 4 (“severely abnormal behavior”). Scores between 30 and 36 indicate mild to moderate ASD, whereas scores between 37 and 60 indicate severe ASD [38]. The reliability and validity of the CARS are well-established, with ICC = 0.80 and Cronbach’s α = 0.82, respectively [39]. Specifically, the CARS 15 items include: “1. Relating to people; 2. Emotional response; 3. Imitation; 4. Body use; 5. Object use; 6. Adaptation to change; 7. Listening response; 8. Taste, smell, touch; 9. Visual response; 10. Fear or nervous; 11. Verbal communication; 12. Activity level; 13. Nonverbal communication; 14. Level and consistency of intellectual response; 15. General impression” [38].

#### 2.3.5. Canadian Occupational Performance Measure

The COPM is a personalized criterion-referenced outcome measure to assess changes in the satisfaction of clients receiving occupational therapy and occupational performance in the ICF model for the participation domain [40]. In the COPM, the client or parents contemplate five problems in their everyday living (for example, dressing, bathing, and feeding) and assess the importance of each problem with a score between 1 and 10. The higher the scores, the higher the satisfaction and performance. The reliability and validity of COPM were previously reported, with ICC = 0.99 and Cronbach’s α = 0.89, respectively [41].

#### 2.3.6. Short Falls Efficacy Scale

The sFES evaluates the subject’s fear of falling in daily living activities in the ICF model for the participation domain. The sFES comprises 7 items. All the scores are summed to obtain a maximum score of 28. Each daily living activity has an ordinal scale with 4 points for each item, ranging from 1 (“not at all concerned”) to 4 (“very concerned”) [42]. The reliability and validity of the sFES were previously reported, with ICC = 0.99 and Cronbach’s α = 0.94, respectively [43]. Specifically, the sFES 7 items include: “1. Getting dressed or undressed; 2. Taking a bath or shower; 3. Getting in or out of a chair; 4. Going up or down stairs; 5. Reaching for something above your head or on the ground; 6. Walking up or down a slope; 7. Going out to a social event” [42].

#### 2.3.7. Pediatric Quality of Life

PedsQL assesses the quality of life of children and adolescents in the ICF model for the participation domain. The PedsQL consists of 23 items on emotional, physical, social, and school functioning. The scores range from 0 (“never a problem”) to 4 (“almost always a problem”). The reliability and validity of the COPM have been established, with ICC = 0.89 and Cronbach’s α = 0.93, respectively [44,45]. Specifically, the PedsQL 23 items include: “1. Walking more than one block; 2. Running; 3. Participating in sports activity or exercise; 4. Lifting something heavy; 5. Taking a bath or shower by him or herself; 6. Doing chores around the house; 7. Having hurts or aches; 8. Low energy level; 9. Feeling afraid or scared; 10. Feeling sad or blue; 11. Feeling angry; 12. Trouble sleeping; 13. Worrying about what will happen to him or her; 14. Getting along with other children; 15. Other kids not wanting to be his or her friend; 16. Getting teased by other children; 17. Not able to do things that other children his or her age can do; 18. Keeping up when playing with other children; 19. Paying attention in class; 20. Forgetting things; 21. Keeping up with schoolwork; 22. Missing school because of not feeling well; 23. Missing school to go to the doctor or hospital” [44].

### 2.4. Intervention

All participants underwent either intervention protocol (CAT or IAT) consistently for 60 min/day, 2 days/week, and 10 weeks.

The CAT in children with ASD comprised stability and mobility exercises in accordance with dynamic or static balance and the SIT was associated with sensory function. In the intervention approaches for the physical domain, physical therapists provided interventions, including hippotherapy [46] and treadmill training [47], based on evaluations and selected clinical evidence. In addition, occupational therapists provided interventions, including SIT [48] for the sensory domain and cognitive behavior modification [49] for the cognitive domain. Intervention for the social integration domain was not implemented in the CAT group.

**Figure 2:**
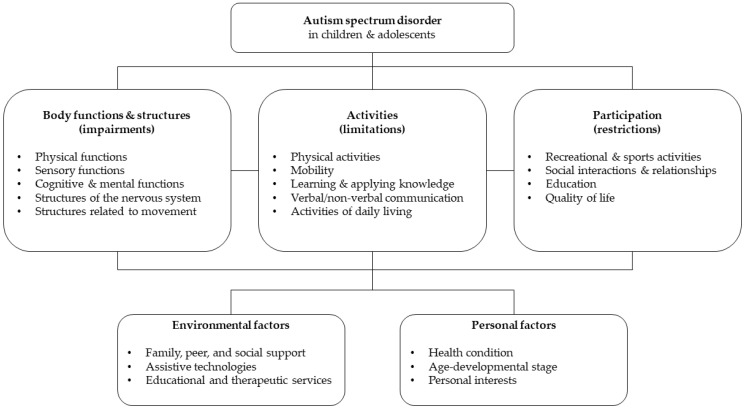
Autism spectrum disorder according to the ICF model.

The IAT applied COPM to identify developmental goals of individual children and adolescents with ASD and to develop specific interventions customized for the gross and fine motor, sensory, cognitive, and social development goals of the children and adolescents based on the four domains (physical, sensory, cognitive, and social integration). These interactions according to the ICF model are described in Figure 2. For each domain, the intervention approaches were formulated by a licensed therapist on the basis of evaluations and selected clinical evidence. Physical therapists provided interventions for the physical domain, including hippotherapy [46], treadmill training [47], and gross and fine motor training. In addition, occupational therapists provided interventions for the sensory domain, including SIT [48]. Occupational therapists provided interventions for the cognitive domain, including cognitive behavior modification [49]. For the social integration domain, occupational therapists executed COPM, cooperated with social worker and the physical therapist for diagnoses, and set goals for the children- and family-centered interventions through a team approach with the parents. Based on evaluations and clinical evidence, the corresponding interventions included counseling regarding the emotional management and quality of life of parents and children or adolescents with ASD and a social integration program in accordance with a focus group interview [50]. The IAT was derived from the integrative framework on the basis of evidence, and the detailed treatment protocol is outlined in Table S1. The established COPM goals and specific intervention approaches were executed at the pediatric rehabilitation center and constantly integrated at the child’s school or community activity center, as well as in the home environment, to maximize physical, sensory, cognitive, and social integration domains.

### 2.5. Statistical Analyses

Statistical data are expressed as means and standard deviations (SD). An independent *t*-test (“equation: $t=\frac{\left({\bar{Χ}}_{1}-{\bar{Χ}}_{2}\right)-\left(\mu_{1}-\mu_{2}\right)}{\sqrt{\frac{s_{1}{}^{2}}{n_{1}}+\frac{s_{2}{}^{2}}{n_{2}}}}$, ${\bar{Χ}}_{1}$ is the mean of first sample, ${\bar{Χ}}_{2}$ is the mean of second sample, $\mu_{1}$ is the mean of first population, $\mu_{2}$ is the mean of second population, $s_{1}$ the SD of first sample, $s_{2}$ is the SD of second sample, $s_{1}$ is the size of the first sample, $s_{2}$ the size of the second sample” [51]) or Chi-square (“equation: $x^{2}=\sum \frac{{\left(O_{i}-E_{i}\right)}^{2}}{E_{i}}$, $x^{2}$ is chi-squared, $O_{i}$ is observed value, $E_{i}$ is expected value” [51]) test was used to compare the baseline clinical characteristics and demographic data between the CAT and IAT groups. A power analysis using G-Power software (G-power software 3.1.9.4; Franz Faul, University of Kiel, Germany) was conducted to assess the sample size on the basis of our previous study, which yielded 24 participants, computing from the effect size (eta squared, η^2^ = 0.6) and power (1 − *β* = 0.8) for the PBS variables. Two-way repeated analysis of variance (ANOVA, “equation: $F=\frac{\sum n_{j}{\left({\bar{Χ}}_{j}-\bar{Χ}\right)}^{2}/\left(k-1\right)}{\sum \sum {\left(Χ-{\bar{Χ}}_{j}\right)}^{2}/\left(N-k\right)}$, ${\bar{Χ}}_{j}$ is the sample mean in the *j*^th^ group, $\bar{Χ}$ is the overall mean, *k* represents the number of independent groups, and *N* represents the total number of observations in the analysis” [51]) was used to compare the following measurements for the pre-tests, post-tests, and follow-up tests: (1) PBS, (2) sSP, (3) FIM, (4) CARS, (5) COPM, (6) sFES, and (7) PedsQL. If an interaction effect was observed, the Bonferroni post hoc test was implemented. All statistical analyses were performed via the Statistical Package for the Social Sciences (SPSS) version 25, software (SPSS Inc., Chicago, IL, USA). The statistical significance level for all tests was set at *p* < 0.05.

## 3. Results

### 3.1. Demographic Characteristics of Participants

All participants who completed the pre-test, intervention (minimum 20 sessions), post-test, and follow-up test were included in the analysis. Table 1 describes the demographic and clinical characteristics of the participants. The demographic and clinical characteristics include sex, age, height, weight, body mass index, intellectual level in CARS, communication (comprehension and expression), and social cognition (social integration, problem solving, and memory) in FIM. There were no statistically significant differences between the CAT and IAT groups in any of the demographic characteristics. Similarly, the baseline parameters for PBS, sSP, FIM, CARS, COPM, sFES, and PedsQL between the two groups did not differ significantly. No safety issues were reported, and none of the participants experienced any side effects associated with CAT or IAT.

### 3.2. Clinical Outcome Measurements

#### 3.2.1. Pediatric Balance Scale

ANOVA showed significant differences in the PBS scores for children with ASD between the CAT and IAT groups (*p* = 0.00) (Table 2). Bonferroni post hoc analysis demonstrated that the IAT group showed a greater increase in the PBS score than did the CAT group (Table 2).

#### 3.2.2. Short Sensory Profile

ANOVA did not show a significant difference in the sSP score between the CAT and IAT groups (*p* = 0.76). However, a significant time main effect was observed in the IAT group (*p* = 0.01) (Table 2).

#### 3.2.3. Functional Independence Measure

ANOVA showed significant differences in the FIM scores between the CAT and IAT groups (*p* = 0.00) (Table 2). Bonferroni post hoc analysis revealed that the IAT group demonstrated a greater increase in the FIM score than did the CAT group.

#### 3.2.4. Childhood Autism Rating Scale

ANOVA did not show a significant difference in the CARS score between the CAT and IAT groups (*p* = 0.15). However, a significant time main effect was observed in the IAT group (*p* = 0.01) (Table 2).

#### 3.2.5. Canadian Occupational Performance Measure

ANOVA revealed significant differences in COPM performance (*p* = 0.05) and satisfaction scores (*p* = 0.02) between the CAT and IAT groups (Table 3). Bonferroni post hoc analysis demonstrated that the IAT group revealed a greater increase in the COPM score than did the CAT group.

#### 3.2.6. Short Falls Efficacy Scale

ANOVA showed significant differences in the sFES scores between the CAT and IAT groups (*p* = 0.00) (Table 3). Bonferroni post hoc analysis demonstrated that the IAT group showed a greater decrease in the sFES score than did the CAT group.

#### 3.2.7. Pediatric Quality of Life

Although ANOVA did not show a significant difference in the PedsQL scores between the CAT and IAT groups (*p* = 0.21), a significant time main effect was observed in the IAT group (*p* = 0.01) (Table 3).

## 4. Discussion

This is a clinical study that highlights the beneficial effects of IAT compared with CAT in the physical (PBS), sensory (sSP), cognitive (FIM and CARS), and social integration (COPM, sFES, and PedsQL) domains in children and adolescents with autism. Consistent with our hypothesis, the IAT group verified a significant improvement than did the CAT group in relation to clinical outcomes in the PBS, sSP, FIM, CARS, COPM, sFES, and PedsQL, which included improved balance, sensorimotor function, cognitive function, autism scale, risk of falling, and quality of life. Most importantly, IAT substantially improved the social integration domain, including occupational performance, satisfaction, fear of falling, and quality of life, which helped individuals with ASD acquire the social integration skills necessary for independent community activities at home and at school. Unfortunately, the lack of clinical IAT evidence in the current literature makes it difficult to compare our results.

The physical domain (PBS) data analysis demonstrated significant improvements in PBS (30%) in the IAT group than in the CAT group. This finding is consistent with the results of a previous study, which demonstrated changes in PBS scores (8.05%) after 12 weeks of therapeutic skating intervention in two boys with ASD [52]. Additionally, another study demonstrated improvement in the Berg balance scale score (62.1%) after 22 weeks of occupational therapy and yoga in nine children with autism [53]. The IAT facilitates rearranging the upright positioning and movement of the pelvic girdle, which helps optimize the tactile, visual, and proprioceptive inputs of the trunk region [54]. This further facilitates discriminatory concentric and eccentric muscle activation of the deep abdominals [55]. The IAT was intended to activate the weak or underactive core muscles and, as a result, consciously and cortically increase tactile awareness of using vibration.

Sensory domain (sSP) data analysis showed a significant time main effect only in the IAT group and failed to produce any meaningful changes between the groups. This finding is consistent with a recent SIT study that demonstrated greater improvements in sensory processing measures in SP scores (between-group mean difference = 0.3 to 9.5) in children with ASD following 12 weeks of SIT [56]. Integration of multisensory stimuli is important for the perception of environmental and social information [57]. A previous electroencephalography study indicated that individuals with ASD do not integrate sensory stimuli in an entirely typical manner [58]. A possible neurophysiological reason for the sensory improvement demonstrated by our study may have been the increased efficiency in unisensory processing of a multisensory trained stimulus [59]. Smith (2019) insisted that stimulating visual sensation in conjunction with other sensory domains (auditory, tactile, and vestibular) could improve an individual’s reactivity to the environment and predictability of tasks [60]. The IAT facilitates multisensory processing through visual (light), auditory (piano), tactile (vibrator), vestibular (hammock, trampoline), and proprioceptive (swing) sensory stimulations.

Cognitive domain (FIM, CARS) data analysis showed significant improvements in FIM (10.5%) and CARS (3.7%) in the IAT group than in the CAT group. This finding paralleled the results of a study which reported changes in CARS (2.77%) after a psycho-educational program in 40 children with ASD [61]. Furthermore, a recent study reported changes in the Wisconsin card sorting test (2.72%) after 12 weeks of physical activity intervention for a group of 22 boys with ASD compared with the control group [62]. A possible primary mechanism for such cognitive domain improvement is that IAT provides knowledge of performance (KP) and knowledge of results (KR) feedback, in which visuomotor learning strategy (clinical and motor) improvements are associated with better comprehension, memory, problem solving, and predictive abilities [63]. These cortical and subcortical cognitive processes are more engaged in acquiring important information about adapting motor controls to improve efficacy. Augmented feedback is widely accepted to facilitate motor skill learning and acquisition. A previous study on augmented feedback indicated that trainers provided feedback, including KR, KP, and behavioral feedback, at a rate of 1.5/min [63]. Furthermore, this study described that over half of all feedback delivered to participants was evaluated in nature and addressed performance outcomes (KR), leaving a smaller percentage of feedback dedicated to information or the execution of a motor pattern (KP) [64]. Mainly, these researchers have applied KP to instruct participants about errors of performance in their motor controls.

Social integration domain (COPM, sFES, PedsQL) data analysis demonstrated significant improvement in COPM (19.8%), sFES (19.7%), and PedsQL (15.6%) in the IAT group than in the CAT group. This finding corroborates the results of Hill et al. (2020), who reported changes in the COPM scores (10%) of 22 children with autism after 9 weeks of canine-assisted occupational therapy compared with those of the control group [65]. A previous study reported an improvement in the COPM score (7.7%) after 12 weeks of occupation-based telehealth coaching in 19 children with autism [66]. The present results demonstrated that IAT can determine which therapeutic approach is most effective for specific children and, therefore, ensure the effectiveness of the intervention through individualized goal-setting and integrative therapeutic programs that meet the specific developmental goals of the participants [67]. Providing parents with more accurate knowledge of their children’s disabilities, along with psychological and emotional support, can encourage more effective use of health and social resources [55]. Maintaining and generalizing these skills and knowledge is important to help families keep their cohesion and harmony over time and to help them to cope effectively with the demanding tasks involved in the education and care of a growing child [68]. Therapists and social workers provide services that take into account children’s abilities, emotional and cognitive skills, and values. This allows parents to be active agents who participate in decisions regarding their children, are conscious collaborators, and are also advocates for their rights and those of their children. Taken together with previous evidence, this study was the first to develop an IAT for children with autism, which may lead to new possibilities concerning an integrated approach to autism therapy, including the physical, sensory, cognitive, and social integration domains.

The limitations of the current study should be addressed in future research. First, the sample size between the groups was not equal because of difficulties in engaging participants due to COVID-19. However, the baseline clinical outcome measurement data were not different between the groups, therefore supporting the assumption of homogeneity; as such, this limitation is unlikely to affect the results. Second, the intervention was temporarily stopped due to COVID-19. This temporary service discontinuity may have influenced the outcomes of our study. The CARS and FIM with observation-based rating scales were utilized due to the majority of participants presenting with low neurocognitive and communication skills, rendering us unable to assess their neurocognitive profiles (e.g., IQ) and adaptive functioning. In the future, more appropriate quantitative measurements of neurocognitive (e.g., the Wechsler Intelligence Scale for Children; the Mullen Scales of Early Learning; the Bayley Scales of Infant and Toddler Development, 3rd edition; and the Psychoeducational Profile, 3rd Edition) and adaptive functioning (e.g., the Vineland Adaptive Behavior Scales, 3rd edition and the Adaptive Behavior Assessment System, 3rd Edition) profiles should be considered depending on what is age-appropriate and communication levels. Nevertheless, our results demonstrated that, compared with CAT, IAT was more effective in multiple domains across the pre-tests, post-tests, and even the 4-week follow-up tests.

## 5. Conclusions

This comparative study demonstrated that, compared with CAT, IAT was more clinically effective in improving physical, sensory, cognitive, and social integration domains in children and adolescents with ASD. Our long-term follow-up findings suggest that the IAT can be an alternative, effective, and sustainable approach to improve multiple domains and to help design appropriate interventions for children and adolescents with ASD by ensuring improved long-term results.

## Figures and Tables

**Table 1:** Demographic and clinical characteristics of participants.

Participation ID	Group	Sex	Age	Height	Weight	BMI	Intellectual Level (CARS)	Communication (FIM)	Social Cognition (FIM)
1	CAT	Male	5	103	20	18.85	4	7	9
2	CAT	Male	4	95	17	18.83	3	8	9
3	CAT	Male	5	108	20	17.14	4	7	11
4	CAT	Female	15	138	29	15.22	2	7	11
5	CAT	Male	12	133	29	16.39	2	7	10
6	CAT	Male	9	148	41	18.71	2	8	10
7	CAT	Male	5	97	26	27.63	4	6	9
8	CAT	Female	9	139	21	10.86	3	10	11
9	CAT	Female	8	130	42	24.85	3	9	11
10	CAT	Male	11	145	35	16.64	5	8	10
Mean		7/3	8.30	123.60	28.00	18.51	3.20	7.70	10.10
SD			3.62	20.60	8.93	4.75	1.03	1.16	0.88
11	IAT	Female	5	103	17	16.02	3	7	10
12	IAT	Male	7	125	22	14.08	3	6	8
13	IAT	Male	6	110	19	15.70	3	8	10
14	IAT	Male	5	100	15	15.00	4	7	10
15	IAT	Female	5	106	17	15.12	4	7	10
16	IAT	Female	14	135	37	20.30	2	8	11
17	IAT	Male	15	152	45	19.47	2	10	12
18	IAT	Male	14	138	34	17.85	3	7	10
19	IAT	Male	7	120	20	13.89	5	6	9
20	IAT	Male	7	120	35	24.30	3	7	9
21	IAT	Male	10	140	41	20.92	2	8	10
22	IAT	Male	8	130	23	13.61	2	8	10
23	IAT	Male	9	140	43	21.94	2	10	11
24	IAT	Male	4	112	24	19.13	2	9	10
Mean		11/3	8.29	123.64	28.00	17.67	2.86	7.71	10.00
SD			3.67	16.08	10.68	3.39	0.95	1.27	0.96
*p*-value		0.14	0.54	0.25	0.09	0.06	0.20	0.87	0.93

Abbreviations: CAT, Conventional autism therapy; IAT, Integrative autism therapy; BMI, Body mass index; Childhood autism rating scale; FIM, Functional independence measure; SD, Standard deviation.

**Table 2:** The outcome analysis on physical, sensory, and cognitive domains.

	CAT Group				IAT Group				*p*-Value	
	Pre-Test	Post-Test	Follow-Up Test	*p*-Value	Pre-Test	Post-Test	Follow-Up Test	*p*-Value	Between Groups	Time × Group
PBS	30.90 ± 11.23	31.80 ± 11.10	32.00 ±11.03	0.75	27.79 ± 11.32	34.57 ±14.15	37.00 ± 13.82	0.00 **	0.76	0.00 **
sSP	134.10 ± 15.21	139.20 ± 19.04	143.70 ± 17.93	0.15	131.79 ± 17.50	138.43 ± 18.35	143.70 ± 17.93	0.03 *	0.87	0.76
FIM	16.80 ± 6.46	17.40 ± 6.31	17.80 ± 6.46	0.01 **	16.57 ± 1.91	18.71 ± 2.33	19.29 ± 2.40	0.00 **	0.64	0.00 **
CARS	36.30 ± 6.43	34.20 ± 6.13	33.40 ± 6.31	0.00 **	37.18 ± 6.24	33.43 ± 5.89	32.82 ± 5.85	0.00 **	0.95	0.15

Abbreviations: CAT, Conventional autism therapy; IAT, Integrative autism therapy; PBS, Pediatric balance scale; sSP, Short sensory profile; FIM, Functional independence measure; CARS, Childhood autism rating scale; * *p* < 0.05, ** *p* < 0.01.

**Table 3:** The outcome analysis on social integration domain.

	CAT Group				IAT Group				*p*-Value	
	Pre-Test	Post-Test	Follow-Up Test	*p*-Value	Pre-Test	Post-Test	Follow-Up Test	*p*-Value	Between Groups	Time × GROUP
COPM										
Performance	3.63 ± 0.95	3.87 ± 0.87	4.06 ± 1.00	0.11	3.30 ± 0.89	4.33 ± 1.30	4.33 ± 1.30	0.00 **	0.76	0.05 *
Satisfaction	3.76 ± 1.37	4.18 ± 1.30	4.20 ± 1.42	0.07	3.39 ± 1.17	4.47 ± 1.22	4.47 ± 1.22	0.00 **	0.90	0.02 *
sFES	10.90 ± 2.51	10.90 ± 2.51	10.90 ± 2.51	1.00	11.21 ± 3.70	9.14 ± 3.39	9.00 ± 3.14	0.00 **	0.38	0.00 **
PedsQL	50.70 ± 13.56	46.80 ± 16.26	45.30 ± 16.11	0.50	49.29 ± 15.31	38.07 ± 14.75	36.29 ± 16.55	0.00 **	0.29	0.21

Abbreviations: CAT, Conventional autism therapy; IAT, Integrative autism therapy; COPM, Canadian occupational performance measure; sFES, Short falls efficacy scale; PedsQL, Pediatric quality of life; * *p* < 0.05, ** *p* < 0.01.

## Data Availability

The data presented in this study are available on request from the corresponding author.

## Supplementary Materials

The following are available online at https://www.mdpi.com/article/10.3390/children9121971/s1, Table S1: Interactive autism therapy protocol [69,70,71,72,73,74,75,76,77,78,79,80,81,82,83].
