# Supplementary material for: Effects of Integrative Autism Therapy on Multiple Physical, Sensory, Cognitive, and Social Integration Domains in Children and Adolescents with Autism Spectrum Disorder: A 4-Week Follow-Up Study
Source: Children (Basel). 2022 Dec 15;9(12):1971. doi: 10.3390/children9121971 (PMC9776954; doi:10.3390/children9121971)
Supplement: Supplementary file 1 [file children-09-01971-s001.zip › children-2062521-supplementary.pdf]

Table S1. Interactive autism therapy protocol

Domains	Purposes	Interventions	Rationale
Physical	Gross motor function<br>-Balance<br>: The ability to provide a stable foundation from which to move.<br>-Skilled mobility<br>: The ability to maintain consistency in performing functional tasks with economy of effort, highly coordinated movement	(1) Hippotherapy with static and dynamic stability (with 10-80 hz and 0.1-2.0 km/h)<br>(2) Treadmill training with dynamic stability (with 0.1-2.0 km/h and gradually altered until the subjects self-selected a comfortable speed)<br>(3) Active weight shift training with gym ball<br>(4) Balance control training with bosu ball	· Hippotherapy: to control using the subconscious feedforward mechanism by which the transversus abdominus and multifidus are synergistically co-activated for upright spinal postural stability [1].<br>· Treadmill training: to promote automatic and rhythmic gait by activating the central pattern generator of the spinal cord and increase coordination of agonist and antagonist muscle in the lower extremities [2,3].<br>· Weight shift training: to coordinate the central nervous system both the postural components that stabilize the body and the prime movement components that relate to the particular motor task [4].<br>· Balance control training: to control the amplitude of muscle activations and to reduce abnormal muscle co-activation [5].<br>·Specific task mobility: to alter elementary locomotor variables in the more advanced stage of motor learning [6].
	Fine motor function	Fine motor skill training including academics, play, self-care skills with piano, pegboard, jelly, toys, clothes, and blocks	· Fine motor skill training: to inhibit abnormal postural reflex and tone and facilitate normal movement patterns and muscle coordination [7].
Sensory	Tactile system	Tactile stimulation with vibrator, toys, and putty.	· Tactile system stimulation: to help the mental and physical framework within an individual's nervous system to properly perceive sensory input, regulate its responses, and understand the significance behind a

			particular, texture, and movement [8].
	Vestibular system	Vestibular stimulation with swing, trampoline, gym ball, and hammock.	· Vestibular system stimulation: to increase in the dendritic arborization and synapses in the hippocampal pyramidal neurons which improves performance in spatial learning tasks [9].
	Proprioceptive system	Proprioceptive stimulation with vibrator and swing.	· Proprioceptive system stimulation: to facilitate mechanoreceptors where muscle spindles were stimulated following synovial tissue stimulation by applying passive proprioception stimulus to the whole body stimulating ventral horn cells through afferent fibers and raising the excitability of alpha motor neurons [10].
Cognitive	Behavior	(1) Behavior modification with positive or negative reinforcement and punishment<br>(2) Habit formation with motivation, ability, and triggers	· Behavior modification: specific responses are performed to occur at different times or that particular reinforcers or punishments may be administered at varying intervals in order to modify behavior [11].<br>· Habit formation: to provide motor learning by performing complexity and repetition of individualized tasks through knowledge of performance (KP) and knowledge of results (KR) based on developmental age of motor learning [12].
	ADL	ADL training including grooming/ personal hygiene, dressing, toileting/ continence, transferring/ ambulating, and eating	· ADL training: to perform specific daily tasks and control the movement of the different segments of the body and the child's patterns of movement and posture to help find ways where the function can be optimized with support needs [13].

Social integration	Emotional, social, school functioning	Focus group interview (30 minutes once each before and after intervention)<br>Counselling (5-10 minutes every weekday)	· Focus group interview: to provide a rich and detailed set of data about perceptions, thoughts, feelings, and impressions of specialist and to find out the people's understanding and experiences about the issue and reasons behind their particular pattern of thinking [14].<br>· Counseling: to help the child achieve his optimal developmental potential by explaining the child's condition and helping the parents to accept it, guiding them regarding proper treatment, addressing concerns about the child and psychosocial stress [15].

* ASD, Autism spectrum disorder; Activities of daily living; ADL.

References

1. Park, J.-H.; You, J.S.H. Innovative robotic hippotherapy improves postural muscle size and postural stability during the quiet stance and gait initiation in a child with cerebral palsy: A single case study. *NeuroRehabilitation* **2018**, *42*, 247-253.
2. Drew, T.; Kalaska, J.; Krouchev, N. Muscle synergies during locomotion in the cat: a model for motor cortex control. *The Journal of physiology* **2008**, *586*, 1239-1245.
3. Pitetti, K.H.; Rendoff, A.D.; Grover, T.; Beets, M.W. The efficacy of a 9-month treadmill walking program on the exercise capacity and weight reduction for adolescents with severe autism. *J. Autism Dev. Disord.* **2007**, *37*, 997-1006.
4. Srinivasan, S.M.; Pescatello, L.S.; Bhat, A.N. Current perspectives on physical activity and exercise recommendations for children and adolescents with autism spectrum disorders. *Phys. Ther.* **2014**, *94*, 875-889.
5. Cheldavi, H.; Shakerian, S.; Boshehri, S.N.S.; Zarghami, M. The effects of balance training intervention on postural control of children with autism spectrum disorder: Role of sensory information. *Res. Autism Spectr. Disord.* **2014**, *8*, 8-14.
6. Fong, S.S.; Guo, X.; Liu, K.P.; Ki, W.; Louie, L.H.; Chung, R.C.; Macfarlane, D.J. Task-specific balance training improves the sensory organisation of balance control in children with developmental coordination disorder: a randomised controlled trial. *Sci. Rep.* **2016**, *6*, 1-8.
7. Azar, N.R.; McKeen, P.; Carr, K.; Sutherland, C.A.; Horton, S. Impact of motor skills training in adults with autism spectrum disorder and an intellectual disability. *Journal on Developmental Disabilities* **2016**, *22*, 28.
8. Puts, N.A.; Wodka, E.L.; Tommerdahl, M.; Mostofsky, S.H.; Edden, R.A. Impaired tactile processing in children with autism spectrum disorder. *J. Neurophysiol.* **2014**, *111*, 1803-1811.
9. Sailesh, K.; Manyam, R.; Jinu, K. Beneficial effects of vestibular stimulation on learning and memory: an overview. *MOJ Anat & Physiol* **2018**, *5*, 212-213.
10. Abbruzzese, G.; Trompetto, C.; Mori, L.; Pelosin, E. Proprioceptive rehabilitation of upper limb dysfunction in movement disorders: a clinical perspective. *Front.*

Hum. Neurosci. **2014**, *8*, 961.

11. Maurice, C.E.; Green, G.E.; Luce, S.C. *Behavioral intervention for young children with autism: A manual for parents and professionals*; Pro-ed: 1996.
12. Sunaryadi, Y. The role of augmented feedback on motor skill learning. In Proceedings of the 6th International Conference on Educational, Management, Administration and Leadership, 2016; pp. 271-275.
13. Weaver, L.L. Effectiveness of work, activities of daily living, education, and sleep interventions for people with autism spectrum disorder: A systematic review. *The American Journal of Occupational Therapy* **2015**, *69*, 6905180020p6905180021-6905180020p6905180011.
14. Dilshad, R.M.; Latif, M.I. Focus group interview as a tool for qualitative research: An analysis. *Pakistan Journal of Social Sciences (PJSS)* **2013**, *33*.
15. Ziolko, M.E. Counseling parents of children with disabilities: A review of the literature and implications for practice. *J. Rehabil.* **1991**, *57*, 29.
